# Genome-wide DNA polymorphism analyses using VariScan

**DOI:** 10.1186/1471-2105-7-409

**Published:** 2006-09-12

**Authors:** Stephan Hutter, Albert J Vilella, Julio Rozas

**Affiliations:** 1 Departament de Genètica, Facultat de Biologia, Universitat de Barcelona, Diagonal 645, 08028 Barcelona, Spain; 2 Department Biology II – Evolutionary Biology, University of Munich, Munich, Germany

## Abstract

**Background:**

DNA sequence polymorphisms analysis can provide valuable information on the evolutionary forces shaping nucleotide variation, and provides an insight into the functional significance of genomic regions. The recent ongoing genome projects will radically improve our capabilities to detect specific genomic regions shaped by natural selection. Current available methods and software, however, are unsatisfactory for such genome-wide analysis.

**Results:**

We have developed methods for the analysis of DNA sequence polymorphisms at the genome-wide scale. These methods, which have been tested on a coalescent-simulated and actual data files from mouse and human, have been implemented in the VariScan software package version 2.0. Additionally, we have also incorporated a graphical-user interface. The main features of this software are: i) exhaustive population-genetic analyses including those based on the coalescent theory; ii) analysis adapted to the shallow data generated by the high-throughput genome projects; iii) use of genome annotations to conduct a comprehensive analyses separately for different functional regions; iv) identification of relevant genomic regions by the sliding-window and wavelet-multiresolution approaches; v) visualization of the results integrated with current genome annotations in commonly available genome browsers.

**Conclusion:**

VariScan is a powerful and flexible suite of software for the analysis of DNA polymorphisms. The current version implements new algorithms, methods, and capabilities, providing an important tool for an exhaustive exploratory analysis of genome-wide DNA polymorphism data.

## Background

The comparative analysis of DNA sequence variation within species (polymorphism) and between species (divergence) is a powerful approach to understand the evolutionary process (e.g. [1,2]), and represents an insight into the functional significance of genomic regions (for instance, see [3]). Particularly, the detection of both positive and negative purifying selection at the molecular level is of major interest. Since positive Darwinian selection is ultimately responsible for evolutionary adaptations, the detection of genomic regions driven by positive selection has profound implications in evolutionary biology as well as in understanding the gene function. The identification of regions evolving by negative selection is also very important as conserved regions are most likely to be functionally significant. The inference of such evolutionary process requires knowing how within-species DNA sequences change under neutrality [4]. In this context, the coalescent theory [5,6] has become the primary framework for the analysis of DNA polymorphism data.

Currently, there are few convincing studies on the action of recent -or ongoing- positive selection at the intraspecific level (e.g. [7-9]). Apparently, the most important difficulty is that demographic events such as migration, population expansions or bottlenecks can mimic the signature of selective processes; therefore, it is not easy to detect the specific imprint of positive selection on individual genes or on short stretches of DNA. The distinction between natural selection and other demographic events requires the surveys of large genome regions (for instance, see [8,10-12]). The detection of negative purifying selection on DNA sequences, on the contrary, has been much easier [13]; in fact negative selection is acting continuously while positive selection is much more episodic. Indeed, there are many surveys where the action of negative purifying selection has been detected even at non-coding DNA regions [e.g. [14,15]]. Undoubtedly, such studies will provide fundamental insights into the functional significance of non-coding DNA. Even so, there are very few studies analysing the within and between-species patterns of nucleotide variation at the genome-wide scale.

Recent genome projects efforts, as the HapMap [16], ENCODE [17], SimYak [18], DPGP [19] and the Mouse Genome Resequencing Project [20] will change radically our capabilities to detect specific genomic regions shaped by natural selection. Although with different goals, these projects will generate SNPs (single nucleotide polymorphisms) data from many whole-genome copies. A limiting critical point has been the absence of adequate bioinformatic tools for such analysis. Although there are powerful programs for molecular population genetic analyses (for instance, ProSeq [21], DnaSP [22] and Arlequin [23]), they are not completely satisfactory for the high-throughput kind of data released by these projects.

Here, we describe version 2 of the VariScan software [24]. In this new version we implemented new methods and features for an exhaustive analysis of DNA sequence polymorphisms at the genome-wide scale, using a graphical user-friendly interface. In particular, the current version of the software allows i) reading several informative-rich genome-wide data files; ii) estimating many population-genetic parameters including coalescent-based statistics; iii) a separate analysis for different genomic regions, functional categories, chromosome locations, etc; iv) adapted analysis for shallow data generated by high-throughput genome projects; v) the identification of relevant genomic regions by using the sliding-window (e.g. [25]) and wavelet-multiresolution approaches [26-28]; vi) the visualization of the results integrated with current genome annotations in the most commonly available genome browsers.

## Implementation

VariScan main algorithms are written in ANSI C. The software also includes a number of scripts written in Perl, and a GUI front-end developed in Java. VariScan currently runs on a wide variety of platforms, such as Linux, MacOS X and Win32. VariScan also uses the LastWave version 2.0 software [29] that is invoked from the Java front-end.

## Results

### New features

VariScan version 2 incorporates substantial improvements over version 1: it implements many new methods and features and also includes a graphical user-friendly interface. Specifically, VariScan 2 allows handling input data files with DNA sequence information from (one or more) outgroup species. This feature allows the current version of VariScan conducting divergence estimates, neutrality tests and other parameters requiring such information. The second major improvement is the possibility to conduct separate analysis of different genomic regions (in exonic, intronic, etc), functional categories (such those defined in the Gene Ontology) and chromosome locations. In addition, VariScan version 2 implements new features to visualize the results of the sliding-window, as well of the wavelet-multiresolution approaches, integrated with current genome annotations in the most commonly available genome browsers. Since the data analysis by using such methods is complicated, we have incorporated an easy-to-use graphical user interface which allows conducting all needed computing steps, including those of the wavelet-multiresolution methods.

### Overview

**Figure 1:**
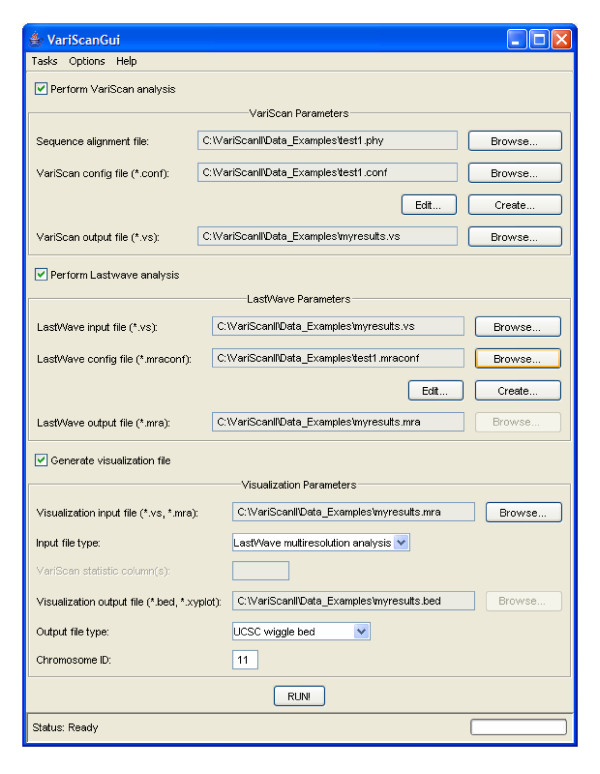
Graphical User Interface of VariScan showing the major options of analysis.

VariScan can read multiple alignment formats as MAF, MGA, PHYLIP, XMGA as those used in the HapMap project [16], with DNA sequence polymorphism data (within-species variation), and also with interspecific nucleotide variation (outgroup information). The software allows conducting exhaustive population-genetic analyses using genome annotations, and permits the visualization of the results integrated in the most commonly available genome browsers. The analysis can be performed using the available GUI (Graphical User Interface) (figure 1) or under a command-line mode.

### Molecular population genetics analysis

VariScan computes state-of-the-art population genetic parameters and coalescent-based statistics including those requiring outgroup nucleotide information [5,6,30,31]. In particular, VariScan calculates (1) the standard summary statistics of nucleotide polymorphism and divergence levels [30,32], such as the population mutational parameter (θ), nucleotide diversity (π), haplotype diversity or the number of nucleotide substitutions per site (*K*); (2) linkage disequilibrium based-statistics: *D*' [33], *r*^2 ^[34], and *Z*_nS _[35]; (3) neutrality-based tests: Tajima's *D *[36], Fu and Li's D*, F*, *D *and *F *[37], Fu's *F*_S _[38], and Fay and Wu's *H *[39]. All parameters and statistics can be conducted by means of the sliding window (SW) [25], or the multiresolution analysis (MRA) approaches [26-28].

#### Missing data

Previous statistics are commonly estimated after excluding all sites with alignment gaps or missing data (i.e., the standard *Complete Deletion *option). However, current genome sequencing projects are generating high-throughput data with a large number of sites with missing information. For example, only ~10% of the polymorphic sites identified in Patil *et al*. [40] study were typed in all 20 chromosomes. Therefore, it is clearly convenient to develop and implement statistics that could capture relevant information included from sites with missing data (about 90% in Patil *et al*.'s data). Here, we have implemented a version of π (π_m_) dealing with missing data. We define π_m _(per site) as



where *l *is the net number of positions surveyed (see below), and *k *is the average number of nucleotide differences that is given by



where *m *is the total number of positions (including sites with missing information, but excluding all positions with alignment gaps), and *h*_*i *_is heterozygosity at site *i*, that is defined as



where *n*_*i *_is the total number of chromosomes (sequences) excluding those with missing data at site *i *(i.e., the net sample size), and *x*_*ij *_is the relative frequency of nucleotide variant *j *(*j *= 1, 2, 3, and 4 correspond to A, C, G, and T) at site *i*. We denote as *l *(the net number of positions) the total number of positions excluding those sites with *n*_*i *_≤ 1. In estimating π_m_, all sites with alignment gaps should be excluded from the analysis. The rationale for this criterion is that while missing data are likely accumulated at random, alignment gaps are not; indeed, two (or more) sequences with gaps in a given position likely correspond to a single insertion/deletion event occurred in a common ancestor.

### Analysis of different functional regions

VariScan allows a fine and detailed analysis of the pattern and levels of nucleotide variation at different functional regions. More precisely, it allows a separate analysis of different genomic regions (e.g., intergenic, noncoding, exonic, intronic, etc.), functional categories (a particular Gene Ontology category), or chromosome locations (specific chromosomal bands or arms, etc.). For the analysis VariScan uses current genome annotations available in public databases. This task is accomplished by a Perl script (*gff2bdf.pl*) that parses the appropriate genome information contained in a GFF (General Feature Format) file [41], and returns a BDF (Block Data File) file directly used by VariScan. The BDF format, which is very similar to that used in VISTA server [42], consists of a tab-delimited list of the relevant positions (the chromosome positions of the genome feature on the reference sequence) to be analysed. *gff2bdf.pl *incorporates several pre-defined filter options; the script, nevertheless, can be easily adapted to accommodate specific or more complex analyses.

### Wavelet transform and multiresolution analysis

VariScan incorporates both the standard SW and the wavelet-based methods to identify particular genome features along the DNA sequence. The wavelet transform (WT), like Fourier transform, is a mathematical transformation widely used to extract information from signals. A signal can be resolved simultaneously in time (or space) and frequency domain by WT. The Fourier transform, on the contrary, only contains frequency information and, therefore, fails to detect spectral components localized in the time (or space) domain. Therefore, wavelet-based analysis provides a method to decompose the signal into high and low frequencies and therefore it is useful in extracting feature information at different scales. For the present analysis, time/space and frequency should be regarded as the position of the nucleotide sequence (a multiple alignment of nucleotide sequence data) and the relevant parameter intensity (levels of nucleotide diversity, linkage disequilibrium, etc), respectively. In this context, the signal is the profile of the relevant statistic along the DNA sequence. Here, we used the WT to decompose the signal into high and low frequencies for detecting global and local relevant features from genome-scale DNA polymorphism data.

There are two basic kinds of WT, continuous (CWT) and discrete (DWT). The CWT of a signal *x*(*t*) is defined as



where τ represents translation (time/space shift), *s *represents scale (or dilation; the inverse of the frequency), ψ(*t*) is the transforming function or mother wavelet, and the asterisk denotes a complex conjugate. There are a number of suitable mother wavelet functions; the choice of the particular mother wavelet to be used, nevertheless, should be adapted to the actual information to be extracted from the signal. Signals are analysed by CWT, which is obtained by scaling and translating (shifting) the mother wavelet along the signal. This process generates the wavelet coefficients (which represent the fit between the function and a particular scale-time of signal) that capture relevant information from a signal.

**Figure 2:**
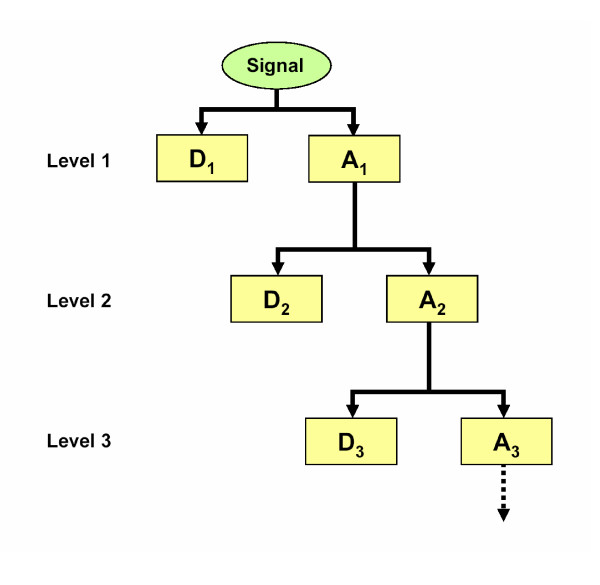
Wavelet decomposition tree. MRA allows for the decomposition of a signal into several resolution levels. First, the original signal (with a power of two points) is decomposed by two complementary half-band filters (high-pass and low-pass filters) that divide a spectrum into high-frequency (detail coefficients; D_1_) and low-frequency (approximation coefficients; A_1_) components (bands). For example, the low-pass filter will remove all half-band highest frequencies. Information from only the low frequency band (A_1_), with a half number of points, will be filtered in the second decomposition level. The A_2 _outcome will be filtered again for further decomposition.

Here, we used the DWT (discrete wavelet transform), which is just the discrete version of CWT, because of the discrete nature of the signal to be analysed (DNA polymorphism data). The signal, which can be envisaged as a one-dimensional vector (of length *L*), is analysed by the *wtrans1d *module of *LastWave v2.0 *software [29] using Daubechies' D4 [43] as the default wavelet filter since it is adequate for locating features, such as peaks and valleys, from a signal [44]. The DWT analysis requires a signal to have a number of points equal to some power of two. For this purpose, and to avoid the boundary effect problem, we used the mirror padding method. With this approach the signal is extended by mirroring both ends at the boundaries, to achieve a total length (*L*') as a power of two. After the WT analysis, the padding tags are discarded and the original signal (of length *L*) is recovered. DWT can be conducted by means of the MRA [27]. This method uses a fast algorithm based on orthogonal wavelets, leading to the decomposition of a signal into different resolution levels; consequently, it enables the extraction of valuable information at different scales. Under this method, the original signal is decomposed by two complementary filters (half-band filters). As a result, the signal is split into two equal parts: one including the high-frequency components (detail coefficients), and the other with low frequency components (approximation coefficients) (Figure 2). While details are not further analysed, the approximation component is successively decomposed, split into two new high and low frequency components. The decomposition process can continue hierarchically until the detail component consists of a single coefficient. Orthogonal wavelets allow for the further reconstruction of the signal, which can be used for an easy location of features along the DNA sequence.

In the context of DNA polymorphism analysis, the signal is the raw profile of the statistic (for instance, nucleotide diversity or linkage disequilibrium levels) obtained along the DNA sequence. The signal is further decomposed to all analysed levels (MRA analysis) using the orthogonal wavelet decomposition method. The orthogonal property of Daubechies' wavelets allows for reconstruction of the signal. The outcome is the reconstructed wavelet-transform profiles of the population genetic parameter along the sequence, which can be used for detecting global and local relevant features (i.e., at different resolution scales) on genome-wide DNA polymorphism data.

### Output visualization

**Figure 3:**
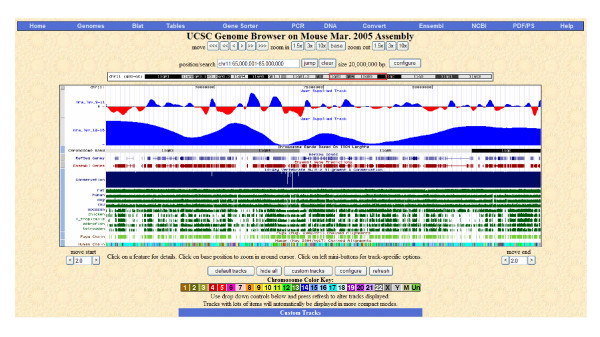
Visualization on the UCSC browser of the MRA analysis based on θ values from the mouse genome resequencing project data [20]. The USCS browser shows a 20 Mb-region (within positions 65.000,001–85,000,000). The first two tracks (customer tracks) represent the signal reconstruction of low-frequency bands with information from 9 to 11 MRA levels (first track), and from 12 to 16 MRA levels (second track).

The SW and MRA results can easily be visualized in available genome browsers (see figure 3), such as the Human Genome Web Browser at UCSC [45] and any Web browser using Gbrowse [46]. This is accomplished by writing the relevant outcome in the so-called custom annotation track formats. In this way, the relevant results (profile of the haplotype or nucleotide diversity along the DNA sequence) can be visualized integrating available genome features (genes, repetitive or intergenic regions, etc).

### Data analysis

We tested the performance of the methods implemented in the VariScan software by analysing two qualitatively different data sets: i) a computer-simulated data set generated by applying coalescent methods, and ii) SNP data from the Mouse Genome Resequencing Project [20], and from the Patil *et al*. [40] study in human. MRA analysis conducted using windows of 1 bp captures all information of the data. Small windows, however, increase the computational RAM-time requirements, and in fact are not strictly necessary. However, we can use larger windows without losing interesting features. Even so, unlike the SW analyses, the MRA results are nearly independent of the chosen window length. Moreover, the SW would likely fail in detecting small-size features at the whole genome scale. For the MRA analysis, the optimal window size to detect most of the interesting features will depend on the current nucleotide diversity values and on of the sample size of the study. These values will be the input (the signal) for the MRA. From a practical standpoint, analysis of 10–30 sequences may be conducted by using non-overlapping windows of 50–500 bp for per-site θ values of 0.01, up to 500–5000 bp for θ = 0.001, as in *Drosophila *and humans, respectively.

#### Computer-simulated data set

**Figure 4:**
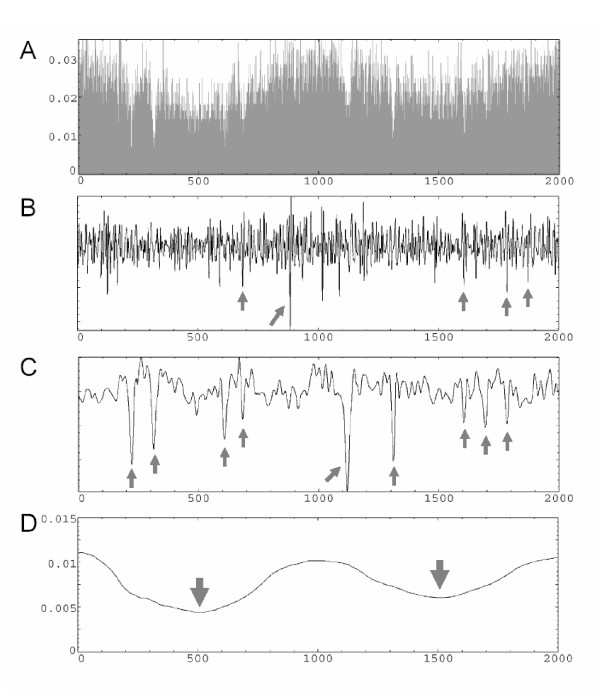
Application of the MRA analysis to the coalescent-simulated data set. The data contains 10 sequences of 2,000,000 bp each, and it was generated applying a per-site value of θ = 0.01. Upon this raw data set, we made two different levels of changes: i) two wide reductions in nucleotide diversity levels (g_1_: α = 1/3, β = 500,000; g_2_: α = 1/2, β = 500,000); and ii) 11 local valleys of reduced variability (v_1_: α = 1/4, β = 20,000; v_2_: α = 1/4, β = 15,000; v_3_: α = 1/4, β = 10,000; v_4_: α = 1/4, β = 5,000; v_5_: α = 1/4, β = 2,000; v_6_: α = 1/3, β = 20,000; v_7_: α = 1/3, β = 10,000; v_8_: α = 1/3, β = 5,000; v_9_: α = 1/2, β = 10,000; v_10_: α = 1/2, β = 5,000; v_11_: α = 1/2, β = 2,000). (a) nucleotide diversity profile obtained by SW using non-overlapping windows of 50 bp; (b) Signal reconstruction of low-frequency bands with information from 7 to 8 MRA levels, showing the location (in arrows) of 5 depleted-variation regions (v_4–5_, v_8_, v_10–11_; β ≤ 5,000). c) Signal reconstruction from 9 to 12 MRA levels, showing the location (in arrows) of 9 depleted-variation regions (v_1–4_, v_6–10_; 5,000 ≤ β ≤ 20,000). d) Signal reconstruction from 13 to 15 MRA levels, showing the location (in arrows) of the two broad areas with reduced levels of variation (g_1–2_; β = 500,000). The nucleotide sequence positions (X axis) are given in kb. .

We generated random data sets based on the simplest non-recombining coalescent model [6] as follows: i) generation of evolutionary times and the gene genealogy (fixing the number of sequences); ii) incorporation of Poisson-randomly distributed mutations (fixing the population mutation parameter θ). Subsequently, we modify this data set by changing (at specific locations) the applied θ value. In particular, we reduced nucleotide diversity values continuously and symmetrically. We made changes at two different levels: iii) one or more chromosome-wide nucleotide diversity reductions; iv) additional reductions at narrow regions. These changes were conducted by using different intensity (parameter α; the degree of nucleotide diversity reduction) and stretch lengths (parameter β; β specifies the half-length of the affected region) values. Therefore, the simulated data set mimics the effects caused by partial selective sweeps upon different nucleotide diversity levels. The analysis of one of these simulated data files is given in Figure 4. It can be seen that the MRA technique recovers the two different intensity types of distorted regions included in the data: nucleotide diversity reductions affecting small DNA stretches are detected at lower MRA levels while more genome-wide reductions are identified at higher levels.

#### DNA polymorphism data from the Mouse Genome Resequencing Project

The Mouse Genome Resequencing Project is conducting a genome-wide DNA resequencing survey in 15 inbred strains of mice using an array-based resequencing technology. In spite that the project in not finished yet, some chromosomes are quite well covered. Here, we use VariScan to analyse the levels of nucleotide diversity along the chromosome 11 (121,803,636 bp; NCBI build 34 which corresponds to the UCSC release of March 2005). Since the polymorphism data were determined in inbred strains (and therefore homozygous) we will consider one sequence per strain (i.e., the sample size is 15). The mouse chromosome 11 data set contains 262,988 SNPs; not all of these SNPs, nevertheless, were typed in all 15 strains because of experimental errors (the average number of missing chromosomes per site was 2.30; and only 91,119 SNPs were typed in all 15 strains). Estimates of nucleotide diversity (π_m_) were π_m _= 0.00072. Nonetheless, since many repetitive regions of the chromosome were not completely resequenced, current nucleotide diversity values likely are underestimated.

Nucleotide diversity values along the chromosome, nevertheless, contain much more information than the global π values. For instance, the SW method allows identifying constrained regions, and it could facilitate the detection of the distinctive fingerprint of positive selection. The MRA analysis is clearly a much more useful method for detecting specific genomic features at different scales. Additionally, the results of these analyses can be visualized integrated with current genome annotations using available genome browsers (figure 3). The MRA analysis revealed a strong heterogeneous nucleotide diversity profile along the DNA region, including a number of peaks and valleys. Although it is premature to determine the evolutionary meaning of these regions, the joint visualization of the MRA results with current annotated genomic features (genes, haplotype information, etc) is a comprehensive tool for their characterization and further understanding.

#### Patil et al. [40] data set

This data set contains the 35,989 SNPs identified in the survey of 32.4 Mb (21.7 Mb after excluding repetitive-masked positions; nearly all human chromosome 21) in 20 ethnically diverse individuals using high-density oligonucleotide arrays. However, for an easy and comprehensible interpretation of the results we do not use this raw data. First, we excluded all singletons variants because the used array-based technology had little power in their identification. Second, we only analysed SNPs confirmed in the NCBI build 34 of the human genome (Patil et al.'s data were based on an older NCBI build). Third, we focused the analysis on SNPs located in the longest contig (NT_002836; named NT_011512 in NCBI build 34 release) of Patil et al.'s data, since there were missing regions between contigs. In total, we analyzed 21,218 SNPs (there were 21,840 in Patil et al.'s data) in a region of 28.6 Mb long (the net number of sites *l *was 19.1 Mb after excluding repetitive-masked positions) in 20 individuals.

For the total NT_011512 contig data, only 2097 SNPs (10%) were typed in all 20 chromosomes, resulting on 3.87 missing chromosomes per site. Estimates of nucleotide diversity (π_m_) was π_m _= 0.00044. This value is lower than that reported in Patil et al.'s study (π = 0.00072) (see also [47]); these estimates, however, are not completely comparable because we are using only a subset of Patil's data. Particularly, we have not taken into account singleton information, while the expected frequency of singletons (mutations occurring on the external branches of the genealogy) for a neutrally evolving region in a sample of 20 sequences is 0.297 (0.321 if we consider that the net number of chromosomes is 16). Thus, roughly 30% of the SNPs should be singletons, although the actual value is likely higher since many human regions have negative Tajima's *D *values. Considering this 30% as the true percentage of singletons in the sample, the π_m _estimates for the total contig would be 0.00050.

## Discussion

Detecting the action of positive natural selection is critical to understand and identify the evolutionary forces that have shaped organismal traits and genomes. Despite the profound implications in evolutionary biology and in medicine currently there are few convincing evidences of the action of positive selection. Since purifying selection weeding out deleterious mutations operates continuously, their detection had been much easier. Indeed, the detection of evolutionarily conserved regions has been proven to be a very effective method for the identification of functionally important regions, such as regulatory elements. The detection of the distinctive signature of natural selection can, nevertheless, be detected by analysing the spatial distribution of polymorphisms across the genome; essentially, positive natural selection causes a distinctive fingerprint on the pattern of nucleotide variation both in the target of selection but also in their surrounding regions. For instance, the selective sweep (or hitchhiking effect) produced when selection drives an advantageous mutation to fixation, will affect variation at relatively short DNA sequence stretches (of some kb; the magnitude of the effect is determined by the relative strength of selection and recombination) [48,49]. On the other hand, demographic effects will have a genome-wide signature.

The identification of the specific regions evolving under natural selection at the genome scale requires, however, new analytical methods and bioinformatics tools. In spite of the impressive recent development of such methods [50], nevertheless, they are not fully adequate for a genome-wide analysis. In this context, VariScan software overcomes many limitations of current software and methods, and it is useful as an exploratory tool in the analysis of DNA polymorphism at the genome scale. VariScan can handle the vast amount of DNA polymorphism data generated by large genome-based projects, and implements efficient methods, such as SW and MRA, to determine the common patterns of nucleotide variation and to identify specific features, along large (chromosome-wide) DNA fragments. The SW has been extensively used in DNA polymorphism studies for exploratory data analysis [22]. This method allows obtaining a relevant parameter profile (e.g., nucleotide or haplotype diversity, linkage disequilibrium) along a DNA region and, therefore, is instrumental in detecting the distinctive footprint of natural selection, mainly in genome wide-based analysis. Unfortunately, the determination of the appropriate window size represents an important limitation of the method. This is a critical point because the accuracy of extracting features from DNA sequence data (i.e, the signature of natural selection) strongly depends on the window size. Although there have been some statistical attempts to determine the window size [51,52], the usual approach is by trial-and-error. The MRA-based analysis, on the contrary, can be used to detect genomic features even at different resolution scales; for example, features in various nucleotide diversity backgrounds. Therefore, the method can be helpful in detecting relevant features from DNA polymorphism data at a genome-wide scale, such as conserved regions, peaks and valleys of nucleotide diversity, linkage disequilibrium clusters, etc. that, in turn, might reveal the distinctive footprint left by the action of natural selection.

## Conclusion

In summary, the version 2 of the VariScan software implements new methods and features for an exhaustive DNA sequence polymorphism analysis at the genome-wide scale. We have tested the performance of the methods implemented in the software by analysing computer-simulated and real data sets.

## Availability and requirements

**Project name**: VariScan

**Project home page**: . Source code, executables and documentation are available from this site.

**Operating system(s)**: Linux, MacOSX, Windows

**Programming languages**: ANSI C, Java, Perl

**Other requirements**: Java 1.4 or higher, Perl 5.6 or higher

**License**: GNU GPL

## Authors' contributions

SH and AJV developed and tested the software. JR conceived and led the project. JR wrote the manuscript. All authors read and approved the final manuscript. SH and AJV equally contributed to this work.
